# Contribution of P2Y_1_ receptors to ADP signalling in mouse spinal cord cultures

**DOI:** 10.1016/j.neulet.2008.02.034

**Published:** 2008-04-25

**Authors:** Amelia E. Atterbury-Thomas, Catherine Leon, Christian Gachet, Ian D. Forsythe, Richard J. Evans

**Affiliations:** a Department of Cell Physiology & Pharmacology, Henry Wellcome Building, University of Leicester, Leicester LE1 9HN, UK; b INSERM, U311, Establissment Francais du Sang (EFS), Alsace, 67065 Strasbourg Cedex, France

**Keywords:** P2Y_1_, P2Y_12_, P2Y_13_, Spinal cord, Calcium, Phosphorylation, Neurons, Glia, ADP

## Abstract

Mixed neuronal and glial cell spinal cord cultures from neonates express ADP sensitive P2Y_1,12&13_ receptors. ADP (10 μM) evoked increases in intracellular calcium that were essentially abolished by the P2Y_1_ receptor antagonist MRS2179 (10 μM), responses were also absent in preparations from P2Y_1_ receptor deficient mice however UTP (100 μM) evoked calcium rises were unaffected. ADP also evoked a robust increase in extracellular signal-regulated protein kinase (ERK) phosphorylation that was of similar magnitude in the cultures from wild type and P2Y_1_ receptor deficient mice. These results suggest that ADP acts through P2Y_1_ receptors to mediate an increase in intracellular calcium but not to stimulate ERK phosphorylation in the spinal cord.

Nucleotides released from neurons, glia and damaged cells play a signalling role in the nervous system. P2Y receptors are a family of G-protein coupled nucleotide receptors, and several of the eight subtypes (P2Y_1,2,4,6,11,12,13,14_) are expressed in the nervous system [11]. P2Y_1_ receptors have been shown to be involved in calcium signalling in a range of neurons and glial cells (e.g. [3,4,9,12]). In addition signalling through recombinant P2Y_1_ receptors stimulates mitogen-activated (MAP) kinases [10] and in native cells P2Y_1_ receptors have been implicated to play a role in extracellular signal related kinases (ERK) activation and stretch induced injury in astrocytes [8]. In this study the aim was to determine whether ADP evoked calcium and MAP kinase responses in cultured spinal cord neurons and glial cells and use knockout mice to determine the contribution of P2Y_1_ receptors to ADP evoked signalling.

Wild type or P2Y_1_ receptor deficient C57 neonatal mice [7] (3–7 days) of either sex were decapitated and the spinal column was removed and washed twice in a calcium/magnesium-free Hanks balanced salt solution (HBSS) containing HEPES (0.3 M), sodium pyruvate and penicillin/streptomycin (50 μg/ml each). The spinal cord was then cut into small pieces and placed in ice cold HBSS. The tissue pieces were then placed in a HBSS-trypsin solution (2.5%) and incubated at 37 °C for 30 min. The tissue was then transferred to a HBSS-trypsin inhibitor solution (1 mg/ml) for 5 min before being replaced with 1 ml Neurobasal media containing glutamine and B27 supplement. Mechanical trituration was used to dissociate the spinal cord cells. The resulting cell suspension was seeded onto poly-d-lysine-coated 16 mm coverslips and maintained at 37 °C in a humidified incubator with a 95% O_2_/5% CO_2_ atmosphere. After 2 h an additional 2 ml of Neurobasal media was added and cells left to grow for 2–5 days.

Spinal cord cultures were ester loaded with a calcium sensitive dye, Fluo-3 acetoxymethyl ester (Fluo-3-AM, final concentration 1 μM) for 30 min at 37 °C in a humidified incubator. The coverslips were then washed in extracellular solution (in mM; 150 NaCl, 10 HEPES, 10 Glucose, 2.5 KCl, 2.5 CaCl_2_, 1 MgCl_2_, pH 7.3) to remove any extracellular dye and maintained at room temperature. Coverslips were mounted in a perfusion chamber on the stage of a Fluoview FV300 confocal microscope and were continuously perfused with extracellular solution. The argon laser excitation of the dye was at 488 nm and the emissions were captured at wavelengths greater than 510 nm by Olympus Fluoview v4.2 software at a frequency of 0.5 Hz for a continuous time period of 280 s. Agonist application was via a U-tube [2]. ADP stocks were treated with hexokinase to remove any ATP contamination. Agonist applications were at intervals of ∼10 min. Antagonists were perfused for 8 min before test solution application so as to allow complete equilibration. Cells displaying ‘puff’ artefacts to just extracellular solution were discounted from analysis [1]. Baseline measurements of fluorescence were calculated as a mean of the 10 s of recording prior to agonist application. Peak changes in fluorescence for neuronal and glial cells were expressed as a ratio of the baseline (increases in fluorescence are expressed as a self-ratio, baseline value is 1).

Spinal cord cells were cultured for 2–5 days as described previously and agonists were applied to individual wells and incubated for 10 min. The cells were subsequently lysed in a solution (in mM; 10 β-glycerophosphate, 1 EDTA, 1 EGTA, 50 Tris–HCl, 1 benzamidine, 1 sodium orthovanadate, 0.2 PMSF, 50 sodium fluoride, 1 mg/ml pepstatin A, 1 mg/ml leupeptin, 0.1% β-mercaptoethanol, 1% Triton X-100). The samples were then mixed with sodium dodecyl sulphate sample buffer (containining β-mercaptoethanol) at a ratio of 1:1 and denatured for 5 min at 95 °C prior to loading on a 10% sodium dodecyl sulphate polyacrylamide gel. The protein was then transferred onto a nitrocellulose membrane and subsequently blocked with blocking medium (5% non-fat milk powder and Tris-Tween 20, buffered salts-TTBS) overnight at 4 °C. Nitrocellulose membranes were then incubated in 5% blocking medium containing either anti-phospho-ERK antibody (Cell Signaling Technology, MA, USA) at a dilution of 1:1000 or anti-non-phosphorylated ERK antibody (Cell Signaling Technology, MA, USA) at a dilution 1:1000. Immunoreactive bands were visualised by incubation of nitrocellulose membranes with anti rabbit peroxidase conjugate at 1:1500 for 2 h at room temperature and then detected with chemiluminiscence (ECL Plus, Amersham, UK) and recorded onto hyperfilm (Amersham, UK). Western blots were analysed using Image J, NIH, USA—http://rsb.info.nih.gov. Phospho ERK intensity was corrected for loading using non-phosphorylated ERK blotting and expressed as fold over basal.

Total RNA was isolated from either intact spinal cord or spinal cord using the RNeasy Mini Kit (QIAGEN, Valencia, CA). The RNA was treated with DNase I (Sigma) to remove any DNA contamination. Half of the purified RNA was reverse-transcribed to cDNA using SuperScript II-reverse transcriptase (Invitrogen, Carlsbad, CA), and the remainder of the RNA was used as a negative control to ensure that there was no genomic contamination. The primers have been described previously [1]. PCR reactions were carried out with 2 μl of cDNA and a final concentration of 500 nM of each primer. Amplification took place in a Techne Genius PCR machine (Techne, Cambridge, UK) using the following protocol: denaturation at 94 °C for 5 min; repeated cycles of denaturation at 94 °C, followed by annealing at 57 °C and extension at 72 °C, each for 30 s; final extension at 72 °C for 10 min. Thirty-five cycles were used. The amplification products were examined by electrophoresis on a 1% agarose gel and visualized using ethidium bromide under UV light.

Statistical tests were preformed using an unpaired *t*-test with a *p*-value of <0.05 considered as significant. Data are expressed as mean ± standard error of the mean.

Spinal cord cells cultured from neonates for 3–7 days contained both neuronal and glial cells that could be distinguished by their morphology and staining with glial fibrillary acidic protein; approx 43% of cells were glia and 57% neuronal (*n* = 576 cells from 4 coverslips). We have previously used calcium imaging to look at P2Y receptor signalling in mouse superior cervical ganglia [1] and similar protocols were used in this study. ADP (10 μM) evoked robust increases in intracellular calcium for both neurons (64% cells responding with peak self-ratio amplitude of 2.20 ± 0.14, *n* = 120 cells from 12 coverslips) and glial cells (57% of cells responded with a peak self-ratio amplitude of 2.17 ± 0.2, *n* = 120 cells from 12 coverslips). Responses were not sustained and generally decayed during the time-course of application (self-ratio at end of 30 s application 1.34 ± 0.08 and 1.27 ± 0.04 for neurons and glia, respectively, *n* = 20 for each).

The selective P2Y_1_ receptor antagonist MRS2179 reduced ADP evoked calcium rises in a concentration-dependent manner and these effects were reversed on antagonist washout (Fig. 1). ADP evoked calcium rises were essentially abolished in spinal cord cultures from P2Y_1_ receptor deficient mice with small residual responses in 11% of neurons (self-ratio 1.14 ± 0.05, *n* = 100 cells from 10 coverslips) and 17% of glial cells (self-ratio 1.21 ± 0.05, *n* = 115 cells from 11 coverslips)(Fig. 2). Responses to the P2Y_2/4_ receptor agonist UTP (100 μM) in cultures from P2Y_1_ receptor deficient mice were the same as for WT for neurons (45 and 48% responding with amplitudes of 1.96 ± 0.09 and 1.79 ± 0.08, *n* = 60, 114 from 6 and 12 coverslips, respectively for WT and P2Y_1_ receptor deficient mice) and glial cells (58 and 62% responding with amplitudes of 1.88 ± 0.13 and 1.67 ± 0.04, *n* = 60, 119 from 6 to 12 coverslips, respectively for WT and P2Y_1_ receptor deficient mice) indicating that Gαq coupled receptor calcium signalling was not affected in the P2Y_1_ receptor deficient mouse (Fig. 2). Taken together these results define a role of the P2Y_1_ receptor in calcium signalling in spinal cord neurons and glial cells. These studies support the role of P2Y_1_ receptors in calcium signalling in the nervous system [3,9,12] and corroborate previous studies using sympathetic ganglion cultures from the P2Y_1_ receptor deficient mouse [1].

P2Y_1_ receptor stimulation has also been reported to mediate ERK phosphorylation in recombinant [10] and native systems [8] and this may contribute to changes in the spinal cord following injury. In spinal cord cultures ADP evoked a time-dependent phosphorylation of p44/42 ERK that was detected at 5 min, peaked at 10 min, and declined back to basal levels by 30 min (Fig. 3A). When just media was added to the cultures there was no change in ERK phosphorylation (Fig. 3B) indicating that ERK stimulation does not result from a mechanical artefact. Adenosine (10 μM) also had no effect demonstrating that adenosine receptors are not involved. We therefore compared ADP evoked ERK phosphorylation in spinal cord cultures from WT and P2Y_1_ receptor deficient mice. Surprisingly ADP (10 μM) evoked similar levels of ERK phosphorylation (∼3-fold increase over basal) in WT and P2Y_1_ receptor deficient cultures (Fig. 3) demonstrating that a receptor other than the P2Y_1_ receptor mediates this ADP response.

Of the currently eight identified P2Y receptor genes ADP is an agonist at Gαq coupled P2Y_1_, and Gαi coupled P2Y_12_ and P2Y_13_ receptors [11]. ADP is also an agonist at the human P2Y_11_ receptor; however a corresponding mouse homologue of the P2Y_11_ receptor has not been detected suggesting that mice do not express P2Y_11_-like receptors [11]. Transcripts for P2Y_1,12&13receptors_ were detected in 3–7 days old spinal cord cultures (data not shown). Amplification of RNA for ADP sensitive P2Y_12&13_ receptors raised the possibility that these may contribute to the ERK phosphorylation. AR-C66931MX [6] is an antagonist of P2Y_12_ and P2Y_13_ receptors with a reported pA_2_ of 8.7 at P2Y_12_ receptors and 0.01 μM reduced ADP responses by ∼80% at recombinant P2Y_13_ receptors (see review [11]). AR-C66931MX (10 μM a supra-maximal concentration at P2Y_12_ receptors) had no effect on ADP evoked ERK phosphorylation indicating that P2Y_12_ receptors did not mediate the ERK phosphorylation. AR-C66931MX may have complex actions at P2Y_13_ receptors, possibly dependent on cell type, and has been described as an activator of P2Y_13_ receptor dependent high density lipoprotein endocytosis in hepatocytes [5] questioning the use of this compound as a selective antagonist at the P2Y_13_ receptor. Thus it remains to be determined whether the P2Y_13_ receptor, or a novel ADP sensitive receptor, mediates the ADP evoked ERK phosphorylation in the spinal cord.

## Figures and Tables

**Fig. 1:**
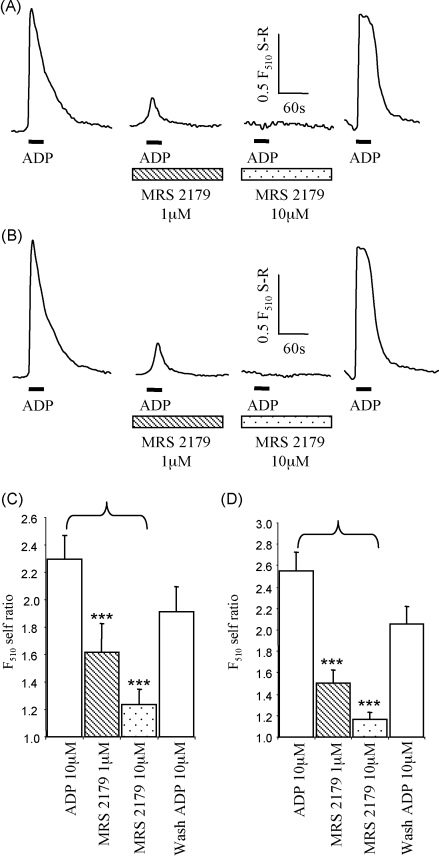
Effects of the P2Y_1_ receptor antagonist MRS2179 on ADP evoked calcium responses from spinal cord co-cultures. (A) Example traces from an individual neuron to ADP (10 μM) and application of MRS2179 (1 and 10 μM) and following washout of the antagonist. (B) Example traces from an individual glial cell to ADP (10 μM) and application of MRS2179 (1 and 10 μM) and following washout of the antagonist. Summary of effects of MRS2179 on neurons (C) and glial cells (D). Data are expressed as the F_510_ self-ratio (F_510_ S-R) of calcium increase compared to basal levels (self-ratio of 1). Drug applications are indicated by bar, *n* = 38 neurons and 45 glial cells.

**Fig. 2:**
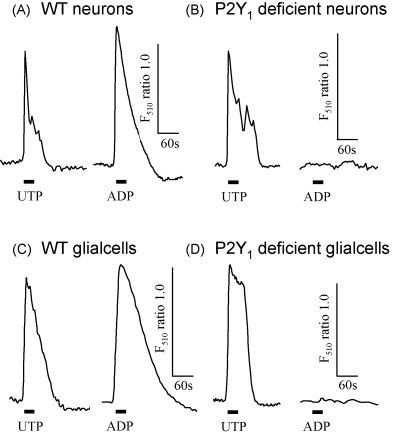
ADP evoked calcium signalling is abolished in spinal cord cultures from P2Y_1_ receptor deficient mice. Sample traces of neuronal responses to UTP (100 μM) and ADP (10 μM) from wild type (WT) (A) and P2Y_1_ receptor deficient mice (B). Sample traces of glial cell responses to UTP (100 μM) and ADP (10 μM) from wild type (WT) (C) and P2Y_1_ receptor deficient mice (D). Data are expressed as the F_510_ self-ratio (F_510_ S-R) of calcium increase compared to basal levels (self-ratio of 1). Drug application indicated by bar.

**Fig. 3:**
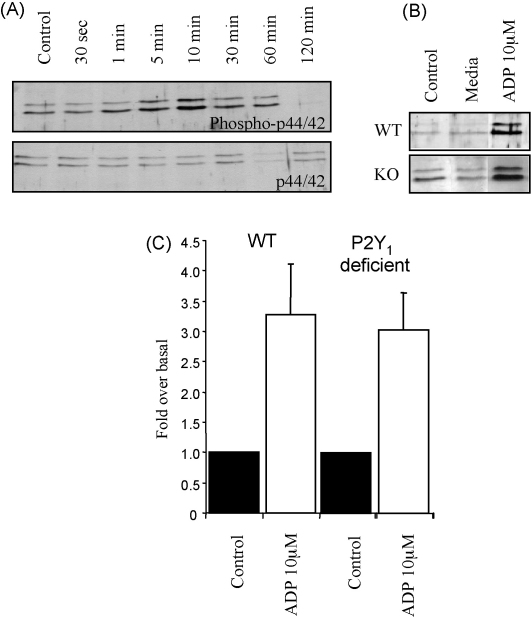
ADP evoked ERK phosphorylation in mouse spinal cord cultures. (A) ADP evoked a time-dependent phosphorylation of ERK p44/42 that peaked at 10 min. Non-phosphorylated p44/42 is shown as a loading control. (B) ADP (10 μM) evoked ERK phosphorylation is observed in spinal cord cultures from both WT and P2Y_1_ receptor deficient (KO) cultures. (C) Summary data for ERK phosphorylation (fold over basal) for cultures from WT and P2Y_1_ receptor deficient mice. *n* = 3 for WT and *n* = 4 for P2Y_1_ receptor deficient cultures.
